# Human mobility and factors associated with malaria importation in Lusaka district, Zambia: a descriptive cross sectional study

**DOI:** 10.1186/s12936-018-2554-4

**Published:** 2018-11-03

**Authors:** Miriam Lowa, Lungowe Sitali, Mwiche Siame, Patrick Musonda

**Affiliations:** 1 Department of Epidemiology and Biostatistics, School of Public Health, University of Zambia, P.O. Box 50110, Lusaka, Zambia; 2 Department of Biomedical Sciences, School of Health Sciences, University of Zambia, P.O. Box 50110, Lusaka, Zambia; 3 Department of Policy and Planning, Ministry of Health, P.O. Box 30205, Lusaka, Zambia; 4 Present Address: Department of Public Health, National Malaria Programme, Ministry of Health and Wellness, P.O. Box 82343, Gaborone, Botswana

**Keywords:** Lusaka district, Malaria importation, Factors, Human mobility, Elimination

## Abstract

**Background:**

Malaria is a major public health problem in Zambia with an estimated 4 million confirmed cases and 2389 deaths reported in 2015. Efforts to reduce the incidence of malaria are often undermined by a number of factors such as human mobility which may lead to introduction of imported infections. The aim of this study was to establish the burden of malaria attributed to human mobility in Lusaka district and identify factors associated with malaria importation among residents of Lusaka district.

**Methods:**

A cross sectional study was conducted in five randomly selected health facilities in Lusaka district from November 2015 to February 2016. Data was collected from 260 patients who presented with malaria and whose status was confirmed by rapid diagnostic test or microscopy. Each confirmed malaria case was interviewed using a structured questionnaire to establish their demographic characteristics, travel history and preventive measures. Travel history was used as a proxy to classify cases as either imported or local. Residency was also used as a secondary proxy for importation to compare characteristics of residents vs non-residents in relation to malaria importation. Logistic regression was used to determine factors associated with malaria importation among residents of Lusaka district.

**Results:**

Out of 260 cases, 94.2% were classified as imported cases based on participants’ travel history. There were 131 (50.4%) males and 129 (49.6%) females. Age distribution ranged from 0 to 68 years with a median age of 15 years (IQR 8–27). Imported cases came from all the ten provinces of Zambia with the Copperbelt Province being the highest contributor (41%). Of all imported cases, use of prophylaxis was found to be highly protective [AOR = 0.22 (95% CI 0.06–0.82); p-value = 0.024]. Other factors that significantly influence malaria transmission and importation by residents include duration of stay in a highly endemic region [AOR = 1.25 (95% CI 1.09–1.44); p-value = 0.001] and frequency of travel [AOR = 3.71 (95% CI 1.26–10.84); p-value = 0.017].

**Conclusion:**

Human mobility has influenced malaria transmission in Lusaka district through a number of factors by importing infections. This leads to onward transmission and poses a challenge to malaria elimination and control. However, taking of prophylaxis is highly protective and must be highly recommended.

## Background

Malaria continues to be a major public health problem globally and one of the leading causes of death from infectious disease worldwide [1]. Global statistics as of 2015 indicated a decline in malaria incidence with 214 million cases and 438,000 deaths of which 88% and 90%, respectively, occurred in the World Health Organization (WHO) Africa region with 97 countries having ongoing malaria transmission [2, 3].

Zambia is a land-locked country with approximately 13.6 million people, 61% of which live in rural areas and 39% in urban [4, 5]. Malaria is endemic throughout the country though it is more prevalent in rural areas. *Plasmodium falciparum* is responsible for most of the malaria cases, including its severe form. In 2015, an estimated 4 million confirmed malaria cases and 2389 reported deaths were due to malaria in Zambia alone [6]. Hence malaria has continued to be a disease of major public health significance in Zambia despite recent successes in scaling up interventions and documented reductions in malaria burden among children [6]. Eliminating infection is, therefore, central to the goal of malaria elimination not only in Zambia but globally. Efforts to attain this goal have been undermined by a number of factors, such as human mobility.

Human population movements play a significant role in malaria transmission [7]. These human movements contribute to the transmission of malaria. The WHO, the United States Center for Disease Control and Prevention (CDC), and most countries define imported malaria as any malaria infection whose origin can be traced to a malaria endemic area outside the country in which the infection was identified. While internal importation is the introduction of parasites from one area to another within a country [3]. To establish the source of infection, knowledge of individual recent travel history is required [8]. The increase in mobility in the last few decades has led to greater concern about the relationship between mobility and malaria transmission. Importation of malaria parasites to low transmission zones from high transmission zones is a major setback in reducing the malaria burden in areas aiming for elimination [9].

There are three malaria transmission zones in Zambia. These are Zone I which includes Lusaka province; Zone II includes Southern, Central, Copperbelt, Western and North-Western provinces and Zone III which includes Luapula, Northern, Muchinga and Eastern provinces [10]. Different trends in the three zones have emerged based on surveys for malaria parasite prevalence in children from 2008 to 2010. Zone I with very low transmission is characterized by parasite prevalence of less than 1% in children under 5 years old; Zone II with low to moderate stable transmission of parasite prevalence of 2–14% in children under 5 years old and Zone III with moderate to high transmission of more than 15% parasite prevalence in children under 5 years old [10]. Seasonal pattern of higher transmission is associated with the rains between November and April. Northern, Luapula and Eastern provinces have the highest annual incidence of malaria, while the lowest is found in Lusaka Province, specifically around Lusaka district.

The burden of malaria is largely attributed to human mobility especially in areas aiming for elimination [8]. Establishing source of infection requires knowing individual recent travel history [8]. Identifying the sources of imported infections due to human travel outside the district and areas of high receptivity within the district aiming for elimination could greatly improve malaria control programmes as this would help target interventions appropriately [11]. This study aimed at establishing the burden of malaria attributed to human mobility in Lusaka district and identifying sources and factors associated with malaria importation among residents of Lusaka district.

## Methods

### Setting

Lusaka province is one of the ten provinces of Zambia with a population of 2,191,225 and density of 100 persons per square kilometre, as of the 2010 census of housing and population. Its capital is Lusaka city which is also a national capital. The study was conducted in five randomly selected health facilities within Lusaka district. These were Chelstone and Kalingalinga health centres with a catchment population of 123,501 and 90,878, respectively; Chawama, Chilenje and Kanyama 1st level hospitals with a catchment population 144,462, 116,510 and 191,056, respectively. These were randomly selected from all health facilities that are run by the government and no private facilities were selected. Health systems in Zambia are classified into three major categories; first level, second level and tertiary level. This selection list included all the health facility levels. A number was given to each facility and place in a bag from which one at a time was picked giving each facility equal chance of being selected. The Zambia Sample Vital Registration with Verbal Autopsy report of 2017 shows that of all deaths that occurred in 2015/2016 attributed to malaria, 86. 6% occurred at government facilities where they received treatment and only 7.4% occurred at private facilities. This indicates that a much larger proportion of the population seeks health care at government facilities compared to private facilities.

### Design, participant selection and data collection

The primary outcome was malaria importation by residents and was defined as a proportion of Lusaka residents who tested positive for malaria and had a travel history to a highly endemic district within a 3 months period prior to the study. Travel was defined as having at least an overnight stay in an endemic area where one could expose themselves to the risk of getting bitten by mosquitoes and consequently getting infected with malaria. Using a cross sectional study design, malaria cases in Lusaka district confirmed either by light microscopy (using 10% Giemsa-stained blood smears examined by two independent examiners), or rapid diagnostic test (RDT) (SD Bioline malaria Ag pf, Standard Diagnostics, INC, Republic of Korea) using manufacturer’s instruction, were utilized.

Primary data was collected from five randomly selected health facilities in Lusaka district. The study population included all confirmed cases of malaria during the data collection period and excluded all clinical cases. Data collection period was limited from November to February and could not be prolonged to the end of the transmission season due to a limited budget. To establish the burden of malaria due to imported cases, the proportion of travellers was determined. This was based on establishing the patients’ travel history. Positive cases that had a travel history within the 3 months prior to the study were considered imported cases. The study investigated internal importation though there were a few cases found to have originated from other countries. A structured questionnaire was administered to all the participants so as to obtain their demographic characteristics, personal protection, travel history and any other information relevant to the study. The interviews were conducted immediately the patient was diagnosed with malaria right at the facility. The obtained information was used to determine the burden of imported cases in Lusaka district, identify the possible sources of infection and factors associated with malaria importation.

### Statistical analysis

Contribution to malaria transmission was determined by assessing factors that influence malaria importation. Travel history was used as a proxy to determine importation and was categorised as either local or imported. Due to the very low numbers of local cases, it was impossible to analyse further using this variable hence Residency was used as a secondary proxy to determine importation as this was effected by both residents with a travel history to highly endemic areas and non-residents from highly endemic areas. Categorical variables were described by frequency distributions. To avoid loss of power and bias, continuous predictor variables such as duration of stay in weeks was not categorized [12]. To test any differences, we used a non-parametric Wilcoxon rank sum (Mann–Whitney) test. Chi square test was used to determine associations of categorical independent variables and outcome variables. Logistic regression was used to explore the association between malaria importation and human mobility. Stepwise logistic regression was used to select the best predictors in a multiple logistic regression model. The association of the individual covariate variables were expressed as odds ratios and their associated p-values. Adjusted odds ratios (AOR) and their 95% CI (p < 0.05) level of significance were reported. All analyses were performed using STATA software, version 12.0 SE (Stata Corporation, College Station, TX, USA).

### Ethical considerations

The University of Zambia Biomedical Research Ethics Committee (UNZABREC) approved the protocol (REF. 003-08-15) and permission was sought from the Ministry of Community Development Mother and Child Health (MCDMCH). Participation in this study was voluntary and the study ensured minimal risk as we enrolled patients who were voluntarily seeking treatment for malaria. For example, the collection of finger prick blood from the patients was a requirement necessary for correct diagnosis and treatment and so it was already done by the healthcare providers. Only individuals who went through the procedure and tested positive were enrolled. Individual consent was obtained from all participants by signing a consent form to acknowledge their participation. Full information about the study was given to the participants. Confidentiality was maintained in line with the local ethical guidelines.

## Results

A total of 260 malaria positive patients were investigated for possible importation of malaria to Lusaka district. The findings of this study showed that 94.2% (95% CI 91.4–97.1%) of the cases investigated were attributed to human mobility and thus classified as imported cases while only 5.8% (15/260) were local cases. This classification was based on participants’ travel history. However, travel history as a primary proxy could not be used further for lack of significant number of local cases. Residency was employed as a secondary proxy for malaria importation and thus compared characteristics of Lusaka residents to those of non-residents with a travel history. Out of a total of 158 Lusaka residents, 15 had no travel history and were excluded from the second stage of analysis, where the factors associated with malaria importation were identified. As such, only those with a travel history including non-residents who obviously had a travel history were analysed at this stage. The median age of the malaria cases was 15 years old (IQR 8–27) with the youngest being less than 1 year old and oldest being 68 years old. Males accounted for 50.4% (131/260) of all cases. Lusaka residents accounted for 61% (158/260) of which 143 had a travel history and thus classified as imported cases (Table 1).

**Table 1:** Background characteristics of cases that tested positive for malaria investigated at selected facilities in Lusaka district, Zambia

Characteristics	Frequency (%) N = 260
Study site	
Chawama	55 (21.2)
Kalingalinga	45 (17.3)
Chilenje	50 (19.2)
Kanyama	60 (23.1)
Chelstone	50 (19.2)
Sex	
Male	131 (50.4)
Female	129 (49.6)
Age group	
(0–4)	39 (15)
(5–14)	81 (31.2)
(15–24)	67 (25.8)
(25+)	73 (28.1)
Median age in years = 15 (IQR 8–27)	
Educational level	
Primary	96 (36.9)
Secondary	82 (31.5)
Tertiary	24 (9.2)
Never been	58 (22.3)
Occupation	
Formal	22 (8.5)
Informal	47 (18.1)
Student	94 (36.2)
Others	97 (37.3)
Residence	
Lusaka	158 (60.7)
Other	102 (39.2)
Travel history (Lusaka residents)	
Travelled	143 (90.5)
Never travelled	15 (9.4)

**Fig. 1:**
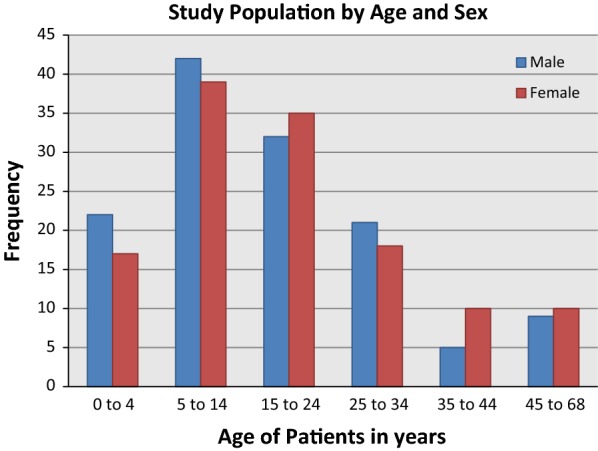
Study population by age and sex

**Fig. 2:**
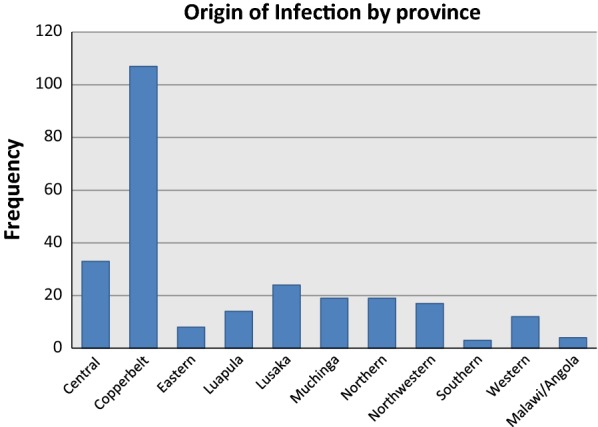
Origin of infection by province as established from patients’ travel history. *Malaria infections whose origin is Lusaka province include Lusaka district and other districts within the province. However, Lusaka district alone had 15 cases which were classified as local cases

The overall study population showed a near balanced representation of males and females. Results showed that the age group 5–14 years was the most affected age group representing 31.2% (81/260) of all cases (Fig. 1). Imported infections were found to be coming from all over the country with Copperbelt Province as the highest contributor (41%) (Fig. 2).

To investigate the association between malaria importation and variables of interest, Chi square test was used and the results established association in the use of prophylaxis, age, duration of stay and occupation. These were found to be statistically significant (see Table 2).

**Table 2:** Association between malaria transmission and predictors of importation among residents of Lusaka district

Variable	Malaria importation by residency		p-value (Chi^2^)
	Residents (n = 158)	Non-residents (n = 102)	
Sex			
Male	80 (51%)	51 (50%)	0.921
Female	78 (49%)	51 (50%)	
Age group			
(0–4)	25 (16%)	14 (14%)	< 0.001
(5–14)	38 (24%)	43 (42%)	
(15–24)	35 (22%)	32 (31%)	
(25+)	60 (38%)	13 (13%)	
Bed net use (always)			
Yes	42 (27%)	27 (26%)	0.984
No	116 (73%)	75 (74%)	
Education level			
Never been	36 (23%)	22 (22%)	0.612
Primary	54 (34%)	42 (41%)	
Secondary	54 (34%)	28 (27%)	
Tertiary	14 (9%)	10 (10%)	
Occupation			
Formal	15 (9%)	7 (7%)	< 0.001
Informal	41 (26%)	6 (6%)	
Student	42 (27%)	52 (51%)	
Others	60 (38%)	37 (36%)	
Prophylaxis			
Yes	4 (2.53%)	10 (9.80%)	< 0.001
No	139 (87.97%)	92 (90.20%)	
Frequency of travel			
Once	122 (85.31%)	93 (91.18%)	0.168
Twice or more	21 (14.69%)	9 (8.82%)	
Duration of stay*	3 (1–4)	1 (0–3)^a^	< 0.0001^b^
Personal protection			
No	133 (84.18%)	86 (84.31%)	0.976
Yes	25 (15.82%)	16 (15.69%)	
IRS (last 12 months)			
Yes	147 (93.04%)	93 (91.18%)	0.582
No	11 (6.96%)	9 (8.82%)	

* Values are medians (interquartile range)

^a^Period less than 1 week to 3 weeks

^b^Two-sample Wilcoxon rank sum (Mann–Whitney) test

Univariate and multivariate analyses were performed using logistic regression. The adjusted predictors of malaria importation were determined by an investigator led stepwise logistic regression (Table 3). The best predictors for the final model were selected for malaria importation among Lusaka residents. After adjusting for other variables such as sex, age and education level; the frequency of travel to other districts was found to be significantly associated with malaria importation. It shows that those who travelled outside Lusaka district more than once were approximately four times more likely to import infections [AOR = 3.71 (95% CI 1.27–10.84; p-value = 0.017)]. Patients who took some anti-malarial prior to their travel had a 78% reduced odds of getting infected and importing infections to Lusaka district [AOR = 0.22 (95% CI 0.06–0.82; p-value = 0.02)]. For every week, increase in the duration of stay in an area visited, Lusaka residents were 25% more likely to import infections [AOR = 1.25 (95% CI 1.09–1.44; p-value = 0.001)] (see Table 3).

**Table 3:** Adjusted predictors of malaria importation by residents of Lusaka district

Variable	Adj. OR (95% CI)	p-value
Frequency of travel		
Once	1	
More than once	3.71 (1.27–10.84)	0.017
Prophylaxis		
No	1	
Yes	0.22 (0.60–0.82)	0.024
Duration of stay (in weeks)	1.25 (1.09–1.43)	0.001

## Discussion

This study established a high proportion of imported malaria compared to local cases in Lusaka district. Despite scaling-up interventions to reduce the incidence of malaria in the district, these imported cases could lead to onward transmission and consequently an upsurge of local cases. Even though this study investigated internal importation, similar studies done in China investigating importation across borders also showed a higher prevalence of imported malaria attributed to human mobility [13, 14]. This shows that human mobility challenges the efforts to attain elimination. Literature has shown that malaria importation is indeed a major factor in malaria transmission not only in low transmission setting but also in high transmission setting [9].

Children aged 5–14 years old were found to be the most affected group and were more susceptible to infection due to their weaker immunity compared to adults, considering the fact that this study investigated only positive cases. Even though children may have been accompanied by adults, the adults may not have been affected in a similar manner due to their stronger immunity. This finding is also consistent with a study by Bradley et al. which established that children aged 2–14 years who had travelled to highly endemic areas were at greater risk of infection and were more likely to import malaria [9]. However, in another study by Li et al., adults aged 21–50 were found to be the risk group. This was attributed to travel due to occupation as it was found to be a factor of importation as the majority of these travelled to endemic regions for work [14].

It was established that taking of prophylaxis among residents of a low transmission zone like Lusaka district was highly protective. This was evident in that residents who took anti-malarial drugs prior to their travel were less likely to import infections. However, some patients were found to be suffering a second episode at the time of the study. This suggests that they were either not effectively treated and were likely to transmit malaria or they suffered from a new infection after being re-exposed as they had visited a highly endemic area. Infected individuals including asymptomatic patients who were not treated prior to their travel were also at risk of importing malaria to low transmission areas hence the need to take prophylaxis. A study by Julio et al. showed that lack of adherence to prophylaxis was a risk factor for malaria infection among members of the Guatemalan contingent deployed to the Democratic Republic of Congo thus leading to importation [15]. Another study by Muehlberger et al. found that travelling without or with ineffective chemoprophylaxis is a major factor for malaria importation [16].

Duration of stay was found to be a factor of malaria importation in Lusaka district. This was statistically significant showing that for every increase in weeks, Lusaka residents visiting highly endemic regions were more likely to import infections. This is because the overall influence on local malaria transmission by residents and visitors depends on the number of infections brought relative to the duration of infection, while contribution to local transmission depends on local receptivity of the place where infections are imported and the duration of stay [17]. The study findings further established that frequency of travel was a factor for malaria importation to Lusaka district. Results show that residents who travelled more than once to highly endemic districts within the last 3 months prior to their diagnosis were approximately four times more likely to import malaria than those who travelled only once.

Origin of infection is one of the factors investigated and from the descriptive analysis; it was found that imported cases came from all over the country with most of these cases coming from the Copperbelt province. However, we cannot conclude that the Copperbelt province has the highest malaria prevalence in the country based on this result but this simply shows that most of these movements were done between Copperbelt and Lusaka, thus making Copperbelt province a major source and Lusaka district a sink for malaria infections. Identifying these sources and sinks would help inform and target programmes to improve prevention and control measures which may lead to malaria elimination.

Indoor residual spraying (IRS) is one of the interventions put in place by the national malaria control programme and the Ministry of Health to combat the spread of malaria. It targets to cover 85% coverage of the households in low to high transmission zones [5]. Results of a Zambia Demographic Health Survey (ZDHS) done in 2011–2013 show that only 12% of households in Lusaka were sprayed. It was established from our study that only about 8% of the cases had their homes sprayed in the last 1 year. According to the WHO World Malaria Report of 2015, such interventions; in Zambia particularly, are funded by the government, global fund and USAID. This shows that it is somewhat dependant on donor funding which could explain the low IRS coverage. Of all the patients investigated, only a quarter of the patients used insecticide-treated nets (ITNs) despite having strong campaigns on the use of ITNs in the fight against malaria. This shows poor uptake of interventions to combat malaria among the locals even if the government plays its role in making such services available. However, the use of ITNs and IRS was not statistically significant to malaria importation in this study but is very relevant in the efforts to control malaria transmission. The relevance of these findings is that low uptake of interventions and preventive measure practice tends to undermine efforts to control malaria with the hope to eventually attain elimination.

The limitations of this study were that the proportion of malaria imported cases could have been undermined due to asymptomatic infections. Pathogens can be introduced into an area at four different stages. This study only looked at importation through infected visitors and through residents visiting endemic regions. Infections which could have been introduced by infected foreign vectors could not be identified and this could have led to overestimation of local cases. Travel history of the patients was used as a proxy to classify cases as imported or local. However, those who were considered as imported cases could still have been infected locally despite having a travel history. The study lacked information regarding the time of travel in relation to onset of illness. Unfortunately, this study could not carry out further tests to show whether one acquired the infection locally or not. The inclusion of a comparator would remedy most of these limitations but the study lacked information on non-malaria cases for a comparator.

## Conclusion

Imported malaria has become a major public health challenge in regions aiming for elimination. The high proportion of imported malaria cases in Lusaka district established in this study suggests that local transmission can consequently go up thus posing a threat to the attainment of malaria elimination. Elimination can only be feasible by implementing control measures based on detecting imported cases, identifying and addressing factors associated with malaria importation so as to control onward transmission. The relevance of this study was that identified sources of infection and the associated factors that influence malaria importation could be targeted and included in vector control interventions and disease prevention programmes.
